# Solly on the Human Brain and Todd and Bowman’s Physiology

**Published:** 1848

**Authors:** 


### Part 2.—Reviews and Notices of New Books.

### ARTICLE I.

*The Human Brain: Its Structure, Physiology, and Diseases.* By SAMUEL SOLLY, F.R.S., &c. Lea & Blanchard: 1848.

*Physiological Anatomy and Physiology of Man.* By B. R. TODD, M.D., F.R.S., and W. BOWMAN, F.R.S., of King's College, London. Lea & Blanchard: 1848.

Among the medical books republished recently in this country are Solly on the Human Brain, and Todd and Bowman's Physiology; both truly valuable scientific and practical works, and both containing an unusual amount of valuable and interesting information relative to the nervous system, anatomically and physiologically considered.

This is a subject, as our readers are aware, to which modern anatomists and physiologists have been directing particular attention of late, and upon which much additional information has been acquired as the result of their investigations; we shall, therefore, take up, in connection with Mr. Solly's work on the brain, so much of the Physiological Anatomy of Todd and Bowman as treats of the nervous system, and endeavor to give to our readers a brief abstract of the views expressed by these authors concerning its structure and functions, with such facts from other sources and remarks as may seem appropriate or interesting in connection with the subject.

All modern anatomists agree in stating that there are two kinds of nervous matter—the corticle, gray or vesicular; and the medullary, white or fibrous.

The vesicular matter, composed of vesicles, irregular in shape, and of a dark color, is found upon the external surface of the cerebrum and cerebellum, in the interior of both the brain and spinal cord, and constituting, in part, the ganglia, both of the sympathetic and sensitive nerves. The white or fibrous matter is the sole constituent of the nerves proper; it is found, also, more or less intimately connected with the gray in the brain, cord, and ganglia.

Anatomical facts, experimental physiology, and pathology, all unite to justify us in coming to the conclusion that the two kinds of matter above named differ from each other very materially, both in their anatomical structure and physiological action.

The vesicular or gray matter is found always constituting a part of those portions of the nervous system in which the true nerves originate and terminate. It is never found connected with other organs, except by means of the white nerve fibre. Its development in any portion of the nervous system is always in proportion to the number of white fibres originating from the part, and to the number and importance of the organs or tissues supplied by them. In view of these facts, most modern authors agree in the conclusion that the manifestations of power as seen acting through the motor nerves upon the muscles, and also the capability of receiving impressions from without through the nerves of sensation, are both dependant upon the vesicular neurine in which they respectively originate and terminate, and which may be supposed to constitute the centers for both the origin and reception of nervous impressions.

The anatomical structure of the fibrous or white substance is very different from that of the vesicular neurine, being composed of numerous ultimate nerve fibres, which are described as so many cylindrical white tubes, filled with a delicately organized soft matter, the axis cylinder. All nerves, whether of motion, sensation, or the special senses, are constituted entirely of these ultimate white fibres, which are found to extend, without giving off branches, or forming connection one with another, from the gray matter of the brain, cord, and ganglia, to all the organs to which nerves are distributed. It is very probable, therefore, that it is the office of the white nerve fibre to transmit nervous influence, whatever may be its nature, to and from the ganglionic centers of gray matter.

It is still a desideratum with anatomists to determine how these ultimate fibres terminate in the gray matter. Some contend that it is in the form of loops, as in the muscles, but the most recent investigations seem to favor the conclusion that each ultimate fibre terminates in a vesicle, and that the substance of the latter is continuous with the axis cylinder of the former.

Messrs. Todd and Bowman make the following statement in reference to this subject: "The fibres of nerves may be traced into the nervous centers, the white or fibrous part of which they contribute to form. As they enter the center, the fibres diverge slightly, either singly or in separate bundles, and pass on to form a connection with vesicular matter in the immediate vicinity of the point of immergence, or at a more remote situation. How the fibres comport themselves with respect to the elements of the vesicular matter is not exactly known. It is certain, however, that nerve-tubes frequently adhere to the sheaths of nerve-vesicles, and that many of them pass between the nerve vesicles, probably to form a connection with more distant ones. This may be well seen in the vesicular matter of any of the centers. It is very distinct in the ganglions, and also sufficiently manifest in the spinal cord or brain. In the last-named centers, some of the tubes which are found in the vesicular matter are reduced to an extremely minute size, and exhibit small varicosities, sometimes at very regular distances from each other.

"Valentin describes a looped and plexiform arrangement of the fibres in the vesicular matter of the centers. Hitherto, such arrangement has eluded our observation so completely, that, but for the high authority on which this statement rests, we should not have deemed it necessary to allude to it. The only confirmation of this view with which we have met is derived from an highly interesting dissection, by Mr. Lonsdale, of Edinburgh, of a monstrosity, in which the spinal cord, medulla oblongata, and cerebellum were absent, but the hemispheres of the brain were present. Several of the encephalic and spinal nerves hung 'as loose threads' in the cavity of the cranium or spine. On examining the free or central extremities of these nerves, their constituent fibres were found to form distinct loops, convex towards the cranial or spinal cavity. These loops were imbedded in granular matter, supposed to be vesicular matter in an early stage of formation. Similar loops were observed by the same anatomist in the cranial nerves of an anencephalous fœtus which had been preserved in spirits."

It is well known that the functions of nerves differ very materially. The motor nerves, for instance, seem to transmit nervous influence from within outwards, and in an opposite direction to that in which impressions are apparently conveyed by the nerves of sensation, it being from without inwards. In tracing the ultimate fibres of the nerves of motion from within outwards, or in the direction of the supposed current, they are found to pass into the muscles and out again in such a manner as to form loops, each constitued of an ultimate nerve fibre which as it is found not to terminate in the muscle, must be supposed to have both its origin and termination in the gray matter of the brain or cord. It seems, then, that the manifestations of the nervous power transmitted by the motor nerves, is not at either extremity of the fibre, but at the point where it passes uninterruptedly through the muscle.

Reasoning from analogy, it seems possible and even probable, that the ultimate fibres of the sensitive nerves terminate in like manner in the form of loops in the gray substance of the brain or cord.

The nerves of motion, sensation and the special senses, are all constituted of white fibres, of apparently the same anatomical structure. The greatest difference between them is in their mode of distribution and termination, and it may be that the difference in their functions is dependant upon this circumstance rather than upon any difference of structure. It is found, for instance, by the recent investigations of Messrs. Todd and Bowman, that branches of nerves are either tactile or gustatory, according as they terminate in one or another of three different varieties of papillæ found upon the surface of the tongue, each indicating by its structure, its appropriate function. Those for touch, for instance, resemble, in every respect, the tactile papillæ upon the ends of the fingers, being protected by an epithelial covering nearly as dense and permanent as the cuticle, and differing very materially from another variety, which are larger, more spongy, imperfectly protected by an epithelial covering, and therefore well adapted in structure for the performance of their appropriate function—the absorption of fluids holding sapid substances in solution.

After describing these different varieties of papillæ very minutely, the authors above named go on with their observations upon this subject as follows:

The reader will at once recognize the broad and obvious distinction between the papillæ last described and all other varieties, and will probably surmise, on structural grounds, that they can scarcely share in the reception of impressions which depend on the contact of the sapid material with the papillary tissue. The comparative thickness of their protective covering, the stiffness and brush-like arrangment of their filamentary productions, their greater development in that portion of the dorsum of the tongue which is chiefly employed in the movements of mastication, all evince the subservience of these papillæ to the latter function rather than to that of taste; and it is evident that their isolation and partial mobilily on one another must render the delicate touch with which they are endowed more available in directing the muscular actions of the organ. The almost manual dexterity of the organ in dealing with minute particles of food is probably provided for, as far as sensibility conduces to it, in the structure and arrangement of these papillæ.

The simple papillæ on the base of the tongue, and those clothing the circumvallate and fungiform papillæ, do not appear to differ from one another in any important structural condition, notwithstanding their variety of outward form and arrangement in the compound organs: their epithelium, though of the scaly kind, is very thin, and would easily permit the transudation of sapid substances dissolved in the mucus of the mouth. The softer and perhaps cellulated interior of these papillæ may have a further influence on the act of sensation, With regard to the use of the singular configuration of the circumvallate and fungiform papillæ, it may be conjectured that the fissures and recesses about their base are designed to arrest on their passage the small portions of the fluids in which the sapid materials are dissolved, and thus to detain them in contact with the most sensitive parts of the gustatory membrane.

We may here allude to a certain gradation that is apparent from the papillæ of touch, through those of taste, to the absorbing villi of the small intestines. Touch shades into taste, and at a lower point sensibility is lost. In the tactile papillæ, the excitant of the nerves merely comes into contact with the exterior of a thick epidermic covering: in those of taste, the epithelium is permeated by the special excitant of the nerves; while the intestinal villi are still more elaborately and exclusively organized for absorption. Another class of papillæ might be here spoken of in conjunction with those of taste, as will be seen at a future page.

*On the precise seat of Taste.*—Authors differ considerably on this subject: some limit it to the hinder part of the tongue about the root and sides; some extend it more or less over the whole dorsal aspect and to the tip; others describe it as existing also on the soft palate; while Magendie is of opinion that the pharynx, gums, and teeth are likewise possessed of it. This contrariety, while it shows the difficulty of the subject, may be in some measure explained by the indefiniteness of tastes when faintly perceived by small portions of the surface, by the influence on taste the commonly associated senses of touch and smell, by some diversities really existing in different individuals, and by the ambiguity necessarily attending experiments on special sensation among the lower animals. As the subject is interesting in its bearing on the question of the nerves of taste, we shall here briefly consider it.

Touch, as it exists in the tongue and other highly endowed parts, discovers to us not merely the presence and physical properties of bodies, but their actual position: we recognize the situation of the impression in reference to the whole organ, by virtue of a power common, in a greater or less degree, to all sensitive nerves. Every one who has attended to the effect of sapid substances applied to small separate parts of the tongue must feel that a similar capacity of assigning the position of flavors accompanies the sensation of taste; and on this power in the nerves of taste, aided, as is usually the case, by the nerves of touch, we greatly rely for the determination of the question before us.

In the first place, all allow that acute taste resides at the base of the tongue, over a region, of which the circumvallate papillæ may be taken as the center, and also on the sides near the base. These parts are supplied solely by the glossial twigs of the glosso-pharyngeal nerves.

Secondly, some writers, among whom are Valentin and Wagner, believe the middle and anterior parts of the dorsum of the tongue to be usually incapable of appreciating flavors; while numerous others hold the contrary opinion, with which our own careful and repeated experiments, on other persons as well as ourselves, quite accord. Sour, sweet, and bitter substances applied to the sides, and especially to the tip of the protruded tongue, we find to be at once distinguished; though, when placed on the middle of the dorsal region, they make little or no impression till pressed against the roof of the mouth. In the latter case, however, the taste of sugar is sufficiently distinct, and referred defiinitely to the spot on which it is laid; so that its being tasted does not depend on its diffusion or removal from the central to the circumferential parts, as some imagine. The region now spoken of is supplied almost solely by the lingual branch of the fifth nerve, though Valentin has described a twig of the glosso-pharyngeal running on the under surface towards the tip.

We conclude generally, with regard to the tongue, that the whole dorsal surface possesses taste, but especially the circumferential parts, viz., the base, sides, and apex. These latter regions are most favorably situated for testing the sapid qualities of food: while they are much less exposed than the central part, to the pressure and friction occasioned by the muscles of the tongue during mastication. The central region, as a whole, is more strongly protected by its dense epithelium, and is rougher, to aid in the comminution and dispersion of the food.

Thirdly, the soft palate and its arches, with the surface of the tonsils, appear to be endowed with taste with various degrees in different individuals. Admirault and Guyat affirm that the sense is acute in a spot about the centre, above the uvula; and in some individuals it has so appeared to us. We have also found evidence of the ex istence of taste on the sides and arches of the soft palate in some individuals, but not on the pharynx, gums, or elsewhere. The soft palate and its arches are supplied by the posterior palatine branches of Meckel's ganglion, and sparingly by the glosso-pharyngeal nerves.

*Of the Nerves of Taste.*—Taste having been shown to exist indepenently in parts supplied, on the one hand solely by the glosso-pharyngeal nerves, on the other solely by the lingual branches of the fifth pair, it follows, as a direct consequence, that these nerves must respectively participate in the sense; and there is, besides, reason to attribute a share to the palatal branches of the fifth. Amid many conflicting, and some quite irreconciliable statements on this disputed point, with which it would be needless to distract the reader's attention, the weight of evidence derived from other sources seems to be much in favor of the above conclusion.

The origin and connections of glosso-pharyngeal nerves, which will be described at a future page, may be referred to in connection with this question. Rapp found no lingual branch ot the fifth in the tongue ot the swan or parrot, both of which have acute taste. The glosso-pharyngeal and par vagum supplied the organ.

It seems, then, that all nerves are constituted of white fibrous matter; and that, so far as can be determined by microscopic observations, the difference in their functions depends upon the mode of distribution and termination of the ultimate fibres, rather than upon a difference in stricture.

Another important office of the fibrous neurine is to form bands or commissures connecting different ganglionic centers of gray matter, as, for instance, the corpus collosum connecting the cerebral hemispheres, the pons varolii uniting the two lateral portions of the cerebellum.

The nervous system of many of the lower tribes of animals is found to be constituted of ganglionic centers of gray matter, connected together by cords of fibrous neurine each forming a distinct nervous center, for the performance of a particular function, and for the origin and termination of nerves passing between the ganglia and supplying organs in the vicinity.

In the nervous systems of the higher orders of animals these ganglionic centers become more numerous and their connctions more intimate in proportion to the perfection of their organization, and to the number and importance of their animal functions, till, in man, their complete union constitutes a continuous chain of ganglionic centers of cineritious neurine, each having its appropriate function to perform, as stated in the following extract from Mr. Solly's work:

This cineritious neurine must be regarded as constituting a chain of ganglia, and not as one continuous ganglion; each set of spinal nerves having its own individual nervous center corresponding to its osseous center or vertebra. It is only by thus regarding the composition of the cord that we can account for the uniformity in the number of the cervical vertebræ in Mammalia; the number of the bones being regulated by the number of nervous centers requiring protection. The reason that these are uniform in Mammalia is, that in this class only is there a perfect muscular diaphragm, with a phrenic nerve, having its specific number of roots and corresponding number of ganglia. The anatomical continuity of this gray matter, and its physiological or functional separation into distinct ganglia or centers, is another fact of importance to show that we must not look for anatomical lines of separation in order to establish distinction of function.

In tracing the spinal cord from below upwards, we find that portion of it within the cavity of the cranium named the medulla oblongata, enlarged and of a complicated structure, and containing a large amount of vesicular neurine which, according to Solly, helps to form six distinct ganglia, three upon each side; the olivary or lingual, for presiding over the movements of the tongue as an organ of speech, and giving origin to the lingual nerve; the lateral or the ganglion of the pneumogastric nerve, and the auditory ganglion.

Mr. Solly's views concerning the functions of different portions of the brain are embodied in the following brief extracts:

*Cerebullum.*—The extensive surface of vesicular neurine which constitutes the ganglionic portion of this encephalic center, shows that it must perform some very important office in the animal economy; that it must, in fact, be a ganglion, or a series of ganglia, of great power. Its extensive nervous and commissural connections also support this opinion. The motor and sensory tracts, as they form the restiform bodies and plunge through the substance of its great transverse commissure, have a connection with its nucleated dynamic vesicle. By the inter-cerebral commissure the cerebellum is intimately associated with the optic, the anterior and posterior cerebral, and the hemispherical ganglia.

From what has been already said in the sections on comparative and human anatomy regarding the function of this organ, the reader will be prepared for my opinion on this subject.

There can, I think, be little doubt but it is a regulator and co-ordinator of muscular action on the one part, most probably by means of the central portion of the cerebellum, viz., the superior and inferior vermiform processes. On the second part, it certainly would appear to hold some relation to the generative function. The pathological and other facts adduced by Drs. Gall, Vimont, and Broussais, on this subject, are very striking, and almost as conclusive as all other physiological evidence.

The *locus niger* in the crus cerebri is the next ganglion for our consideration; it is the serial homologue and analogue of the anterior peaks of gray matter of the spinal cord. It is, I suppose the seat of the excito-motor power of the third pair of nerves, the importance of which, in relation to the instinctive and conservative movements of the eyeball need not be insisted on here.

The *tubercula quadragemina*, or optic tubercles, we may fairly conclude, are the instruments by which the physical impressions of light received by the retina are converted into sensations of light, color, form, &c.

The optic thalami and corpora striata, or anterior and posterior cerebral ganglia, are the next in rotation. With regard to the office of these nervous centers, we have already had occasion to consider the thalamus as the essential ganglion of the sensory tract, as the corpus striatum is of that of the motor tract.

We come lastly to those important ganglia which crown and cover in the rest—the hemispherical. If there is one point in the physiology of the brain more unequivocally demonstrated than another, it is that these ganglia are the instruments of the mind—the portions of the brain in which sensations are converted into perceptions, and give rise to ideas. Comparative anatomy; developmental anatomy; experiments on living animals; observations on its size and form, as indicated by the size and form of the skull; and last, but not least, pathology—all afford a mass of overwhelming evidence that this portion of the brain, and this only, is the cerebral organ of intellectual power.

In comparing the two works before us, so far as both treat of the structure and functions of the brain and nerves, it will be found that Mr. Solly is disposed to adopt the popular theoretical views of others, to indulge in speculations of his own, and, in some instances to claim originality in the publication of views and opinions long since expressed by others. If we are capable of judging, Messrs. Todd and Bowman have taken a different course, and one better adapted for the advancement of science. Their physiological deductions are based principally upon well established anatomical facts, supported by the experiments of others, verified by themselves, and by a judicious selection of known facts in pathology, illustrative of physiological action. Theoretical views of their own and of others appear to be considered by them as valuable only so far as they may lead to the discovery of new truths.

In order to show our readers that we are justified, to some extent at least, in making these remarks, we will direct their attention to a part of the quotation last made from Solly's work, in which he unhesitatingly adopts the phrenological views with regard to the functions of the cerebellum, and also to the following, in our opinion, conclusive objections made by Todd and Bowman to this, at one time, popular theory:

It remains for us to notice the celebrated theory of Gall that the instict of propogation has its seat in the cerebellum; which, indeed, according to the author of the theory, and the majority of his followers of the phrenological school, is exclusively devoted to that function. We conceive that this view is far from admissible, on several grounds, of which the following deserve particular mention.

1. It is extremely questionable how far the sexual instinct admits of being separated from the emotions—from those especially which are clearly instinctive in their nature; and, even if it were separable from them, its seems scarcely of such importance, when compared with the other instincts, as to need a separate organ of great magnitude and of complex structure. If we compare it, for example, with the instict of self-preservation, as manifested in providing either for the wants of the body, or for defence against assault, it certainly cannot be admitted to have a superior influence in the animal economy to this, the most pressing of all. Yet it is not pretended to assign a separate seat, even to this.

2. The nature of the generative instinct is scarcely such as to require in its central organ connections so extensive as those possessed by the cerebellum. It is not likely that this organ would be connected with any other part of the spinal cord than that from which nerves are derived to the organs of generation; nor is it conceivable that an instinct like this should require for its exercise fibrous matter in such large quantity as exists in the cerebellum, taking its rise from so great a surface of vesicular matter.

3. The generative instinct is not so pre-eminently developed in man as to account for the great superiority in size, as well as structure, of the human cerebellum over that of the lower animals, even of the mammiferous class. On the contrary, it may be safely asserted that this instinct is much more powerful in the monkeys, and also in the frogs; in the latter of which the cerebellum is absolutely very small, and especially so, relatively to the spinal cord and the cerebral lobes.

4. If the cerebellum be the seat of the generative instinct, it ought to have exhibited marked indications of wasting, in cases where the genital organs have been mutilated; or where they have decayed in the natural progress of age. Yet the recorded cases of this nature are by no means conclusive; on the contrary, M. Leuret's remarkable observations show, that, in the gelding, the cerebellum is actually heavier than in either the stallion or the mare.

5. It does not appear, from pathological research, that the cerebellum has any peculiar influence upon the genital organs. Injury or disease of that organ very rarely produces any effect upon the penis; but lesion of the medulla oblongata or of the spinal cord is very apt to occasion a semi-erection of that organ.

The hypothesis of Dr. Hall with regard to the so called excito-motory system of nerves, reflex action, &c., are evidently regarded by Mr. Solly as well established truths in anatomy and physiology, about which there can be no question or difference of opinion among medical men. "These important discoveries," he remarks, "have been passed by unnoticed and unknown by that scientific society whose object is to promote science and reward real merit." This admission that the Royal Society has not as a body adopted or even taken notice of Dr. Hall's hypothesis is sufficient evidence of its not having been so generally adopted as we would be led to suppose by our author's remarks, and it seems to us that until this is the case this or any other mere theory, however attractive it may be, should be treated of as hypothetical, especially in such as are intended to be standard works, and to treat of important subjects like that of the anatomy, physiology, and pathology of the brain.

By the following quotation from the work of Todd and Bowman it will be seen that there are at least two other theories, with regard to nervous action, equally as plausible as that of Dr. Hall:

What is the *mechanism* of these actions of the spinal cord—mental, physical, locomotive? This is a problem of the highest interest, bearing upon the mechanism by which nervous power developed in any nervous center, as well as in the cord, is capable of affecting peripheral parts; and it is on that account well deserving the most patient investigation.

We assume, as necessary postulates, preliminary to the discussion of this question, the two following propositions: 1. That the brain, or some part of it, is the sensorium commune; or in other words, that mental nervous actions (acts of volition and sensation) cannot take place without the brain. 2. That the vesicular is the truly dynamic nervous matter, the source of all nervous power,

The following hypotheses have been proposed in explanation of these actions.

1. The various muscles and sentient surfaces of the body are connected with the brain by nerve-fibres, which pass from the one to the other. Those fibres destined for or proceeding from the trunk to the brain pass along the spinal cord, so that that organ is in great part no more than a bundle of nerve-fibres going to and from the brain. These fibres are specially for sensation and voluntary motion.

But, in addition to these, there is another class of fibres proper to the spinal cord and to its intra-cranial continuation, which form a connection with the gray matter of the cord. Of these fibres, some are afferent or incident, others efferent or reflex, and these two kinds have an immediate but unknown relation to each other, so that each afferent nerve has its proper efferent one, the former being *excitor*, the latter *motor*. The aggregate of these fibres, together with the gray matter, constitutes the *true spinal*

*cord* of Dr. Marshall Hall, which is not limited to the spinal canal, but passes up into the cranium as far as the crura cerebri. These fibres are quite independent of those of sensation and volition, and of the sensorium commune. Although bound up with sensitive and motor fibres, they are not affected by them, and they maintain their separate course in the nerves as well as in the centers. Such is the hypothesis of an *excito-motory system of nerves*, and of a *true spinal cord*, the center of all *physical* nervous actions, which has been proposed and most ably advocated by Dr. Marshall Hall.

2. The fibres of sensation and volition proceed to and from some part or parts of the intra-cranial mass. Those which are distributed to the trunk pass along the spinal cord, separating from it with the various roots of the nerves, and in the course within the spine they mingle more or less with the gray matter. There are no other fibres but these (save the commissural), and they are sufficient to manifest the physical as well as the mental acts. Nerves of sensation are capable of exciting nerves of motion which are in their vicinity; and they may produce this effect even when the spinal cord has been severed from the brain, for their relation to the gray matter of the cord is such that their state of excitement is readily conveyed to it. This explanation tallies with the views of Whytt, Prochaska, and the other physiologists who had recognized the existence of a class of actions produced by the influence of sensitive upon motor nerves,

3. According to a third hypothesis, it is assumed that all the spinal and encephalic nerves, of whatever function, are implanted in the gray matter of the segments of the cerebro-spinal center with which they are severally connected, and do not pass beyond them. The segments are connected with each other through the continuity of the gray matter from one to another, and through the medium of commissural fibres which pass between them. Through these means, motor or sensitive impulses are propogated from segment to segment; and a stimulus conveyed to any segment from the periphery may either simultaneously affect the brain and cause a sensation, or be reflected upon the motor nerves of that segment and stimulate their muscles to contract.

The phenomena of nervous action, as our readers will perceive, can be explained by either of the above theories; but in adopting that of Dr. Hall, we are obliged to assume what anatomists have not as yet been able to demonstrate, viz., that each compound nerve is constituted of four orders of fibres—nerves of sensation and of volition, nerves for conveying imperceptible impressions, and others for transmitting the stimulus causing involuntary contractions; and that the two first are in connection with the brain and the two last with the cord or other portions of the nervous centers not directly concerned in perception or volition.

The third hypothesis assumes what is more nearly in accordance with known anatomical facts, that the nerves both of sensation and of motion, terminate in or originate from the gray matter of the cord, and that the effect of impressions made upon these ganglionic centers through the sensitive nerves, may be transmitted by means of the fibrous connections with both, to the muscles or brain, to produce either involuntary motion or sensation; and also that the gray matter giving origin to the motor nerves responds, both to the stimulus of external impressions conveyed from without by the nerves of sensation, and to the mental mandate transmitted from within by the white fibres of the cord, to produce either involuntary or voluntary motions.

It seems to us that this theory is much less objectionable than that of Dr. Hall, from the fact that its plausibility depends less upon the assumption of anatomical facts; it is less complicated and therefore more nearly in unison with the simplicity of nature's operations.

It supposes that the gray matter from which the motor nerves originate, may be excited to action by either of two kinds of stimulus, that is, by impressions made upon the sensitive nerves or by mental action transmitted from the brain. There are numerous instances of the kind in which organs are excited to action in different ways. The muscles, for instance, contract under the influence of mechanical irritation, electricity, or the nervous stimulus. The salivary glands are excited to action by the presence of irritating substances in contact with them, or by stimulus transmitted through the nerves and brain from the eye by the sight of food; and in fact it may be said to be a general rule with regard to the performance of the animal functions, that the same effects are most generally produced by a variety of causes.

Having shown our readers, as we believe, that, in the present state of knowledge concerning the structure and functions of some portions of the nervous system, it would be absurd to adopt, without qualification, either of the above or any other hypotheses to explain nervous action, we will make one other short quotation from Mr. Solly's work in order to show with what confidence he adopts hypothetical notions.

The spinal nerves are connected with the spinal cord by anterior and posterior roots. Each root consists of two sets of nerves, making, therefore, four sets of spinal nerves functionally distinct. The two anterior are the conductors of volition from the brain to the voluntary muscles, and the conductors of a stimulus to muscular action independent of volition from the ganglia of the spinal cord—the efferent nerves of spinal power. The posterior roots are also binary in their functional power—nerves of sensation conducting impressions to the brain, and recognized by the conscious being; and conductors of impressions to the spinal ganglia from parts requiring the protective action of muscles too important to be left to the control of mind—the incident nerves of spinal impressibility.

It will be seen by the above extract, that the author seems to take it for granted that four orders of nerve fibres have been discovered; that their functions have been determined; and that a nervous current is known to exist, passing always from without inwards in the sensitive, and from within outwards in the motor nerves.

With regard to the two first suppositions, it may be said, without the possibility of being thought incredulous, that it is still a desideratum to determine for certainty what are the facts; and as to the passage of a current in certain directions it may be remarked that, although it is true that most authors speak of such a current as if no doubt could be entertained of its existence, still we are not justified by facts in considering this anything better than a theoretical view of the subject, adopted to facilitate the explanation of nervous phenomena.

In conclusion, we may remark that the views almost universally adopted with regard to the nervous current, is, as just stated, that it passes from the nervous centers to the muscles to produce contractions. Matteucci, on the other hand contends that electricity is constantly being developed in muscles, rendering them positive; that the relaxed condition depends upon the repulsive power thus existing in the ultimate particles, and that contractions are caused by electrical discharges taking place along the motor nerves in the direction from without inwards, consequent upon and controlled by acts of volition. H.

